# Editorial: Structure Related Druggability of Voltage-Gated Sodium and Calcium Ion-Channels to Treat Diseases

**DOI:** 10.3389/fphar.2022.947511

**Published:** 2022-06-15

**Authors:** Tamer M. Gamal El-Din, Thomas Zimmer, Mohamed Chahine

**Affiliations:** ^1^ Department of Pharmacology, University of Washington, Seattle, WA, United States; ^2^ Institute of Physiology, University Hospital Jena, Friedrich Schiller University, Jena, Germany; ^3^ Department of Medicine, and CERVO Brain Research Centre, Laval University, Québec, QC, Canada

**Keywords:** druggability of voltage-gated sodium channels, drug design, VSD, fenestrations, pore domain

Voltage-gated ion channels play crucial roles in both excitable and non-excitable cells. Therefore, they constitute interesting drug targets. About 15–20% of the drugs used to treat human diseases target ion channels (Overington et al., 2006). High blood pressure calcium channel blockers, antiarrhythmics, antiepileptics, and local anesthetics, which target voltage-gated sodium channels, are all examples of drugs that target voltage-gated ion channels. Most of these drugs use the same mechanism in inhibiting ion channels’ activity, which is blocking the central pore (Gamal El-Din et al., 2018). A small number of medications target other areas of the channels and allosterically enhance the collapse of the pore (Tang et al., 2016; Zhao et al., 2019). The main disadvantage of pore blocker drugs that target voltage-gated sodium and calcium channels is that they are not specific for one type of voltage-gated ion channels. This may engender serious side effects that sometimes can be fatal. So, the aim is to develop a drug that specifically targets one type of a voltage-gated ion channel with high specificity and low toxicity that would be beneficial to patients who suffer from drugs’ side effects. The purpose of this Frontiers in Pharmacology Research Topic is to shed light on the subtle structural differences between different isoforms of voltage-gated sodium and calcium channels, which could be used as a marker to target one subtype of each ion channel. It will also help medicinal chemists, biophysicists, and structural biologists interested in structure-based drug design to use these differences into their considerations during the rational drug design process. We also aimed to focus on discussing the potential druggability of each structural component of the voltage-gated sodium and calcium channels. Recently, new channels’ areas have been proposed to play a role in channelopathy, drug access and binding (Gamal El-Din et al., 2018; Gamal El-Din and Lenaeus). In this Research Topic, Wisedchaisri and Gamal El-Din explored in their review classical drug receptors in voltage gated sodium and calcium channels that have recently been structurally characterized by cryo-electron microscopy with natural neurotoxins and clinical drugs (Clairfeuille et al., 2019; Xu et al., 2019; Jiang et al., 2021; Wisedchaisri et al., 2021). They also examined recent drug discoveries for voltage-gated sodium channels and discuss opportunities to use distinct, state-dependent receptor sites in the voltage sensors as unique drug targets. In the final part of their review, they explored potential new receptor sites that are currently unknown for sodium channels but may be valuable for future drug discovery. On the other hand, Nguyen and Yarov-Yarovoy focused on current challenges and future opportunities to target pain sodium channels (Na_v_1.7, Na_v_1.8, and Na_v_1.9) with small molecules and peptides to design highly potent and selective Na_V_ inhibitors. They explored the strengths of using computational tools like Rosetta and Alpha fold in structural guided development of pain therapeutics. Körner et al. reviewed the history of local anesthetics (LAs) to layout new perspectives in the use of these drugs clinically and in basic ion channel research. They also provided insights into different modelling approaches that will help to identify the exact molecular binding orientation, access pathways and pharmacokinetics (O’Leary and Chahine, 2018).

Understanding how tumor therapeutics interact with voltage gated sodium channels was the topic of the research article presented by Fuchs et al. They showed that imipridone TIC10/ONC201, which belongs to a novel class of anti-cancer compounds, interacts with the Na_v_1.5 cardiac sodium channel, which is found to be expressed in a variety of diverse malignancies including breast cancer, colon cancer, ovarian cancer, melanoma, astrocytoma, or neuroblastoma. They found that TIC10/ONC201 acts as a strong blocker of Na_v_1.5 and thus might contribute to the anti-tumor activity of the drug. In the same direction of looking at the side effects/novel mechanisms of using some drugs to treat certain diseases, Föhr et al. found that atomoxetine, a neuroactive drug, approved for the treatment of attention-deficit/hyperactivity disorder (ADHD) blocks human cardiac sodium channel Na_v_1.5 with high affinity. They reasoned that this could be the cause of sudden death that occurs with several psychotropic drugs. In contrast, Labau et al., elucidated the mechanism by which lacosamide, an anti-epileptic drug that has been used recently to treat pain, interact with pain sodium channel Na_v_1.7. They suggested a hybrid mechanism of inhibition of Na_V_1.7 by therapeutically achievable concentrations of lacosamide where the drug must interact with a specific residue on the VSD4, W1538, before binding in the pore.

Long QT syndrome is a life-threatening genetic disease. Most of the mutations that occurs in the cardiac sodium channel Na_v_1.5 lead to LQT3 or Brugada syndrome (Zimmer et al., 2014). Cano et al. investigated the potential use of ranolazine in treating specific LQT3 disease caused by a specific mutation, V411M, instead of using flecainide. Using Markovian sodium channel model, they compared the efficacy of using ranolazine and flecainide in affecting V411M mutation model. They concluded that patients with LQT3 caused by the heterozygous V411M mutation could benefit from a treatment with ranolazine rather than flecainide.

Despite that structure-based drug design became an essential and invaluable tool for faster and more cost-efficient development way of new medications compared to traditional methods, it is still in its infancy and needs more work to get different structures of the same ion channel at more than one physiological state.

## References

[B1] Clairfeuille T. Cloake A. Infield D. T. Llongueras J. P. Arthur C. P. Li Z. R. (2019). Structural Basis of α-Scorpion Toxin Action on Nav Channels. Science 363, eaav8573. 10.1126/science.aav8573 30733386

[B2] Gamal El-Din T. M. Lenaeus M. J. Zheng N. Catterall W. A. (2018). Fenestrations Control Resting-State Block of a Voltage-Gated Sodium Channel. Proc. Natl. Acad. Sci. U. S. A. 115, 13111–13116. 10.1073/pnas.1814928115 30518562 PMC6304959

[B3] Jiang D. Tonggu L. Gamal El-Din T. M. Banh R. Pomès R. Zheng N. (2021). Structural Basis for Voltage-Sensor Trapping of the Cardiac Sodium Channel by a Deathstalker Scorpion Toxin. Nat. Commun. 12, 128. 10.1038/s41467-020-20078-3 33397917 PMC7782738

[B4] O'Leary M. E. Chahine M. (2018). Mechanisms of Drug Binding to Voltage-Gated Sodium Channels. Handb. Exp. Pharmacol. 246, 209–231. 10.1007/164_2017_73 29138928

[B5] Overington J. P. Al-Lazikani B. Hopkins A. L. (2006). How Many Drug Targets Are There? Nat. Rev. Drug Discov. 5, 993–996. 10.1038/nrd2199 17139284

[B6] Tang L. Gamal El-Din T. M. Swanson T. M. Pryde D. C. Scheuer T. Zheng N. (2016). Structural Basis for Inhibition of a Voltage-Gated Ca(2+) Channel by Ca(2+) Antagonist Drugs. Nature 537, 117–121. 10.1038/nature19102 27556947 PMC5161592

[B7] Wisedchaisri G. Tonggu L. Gamal El-Din T. M. McCord E. Zheng N. Catterall W. A. (2021). Structural Basis for High-Affinity Trapping of the NaV1.7 Channel in its Resting State by Tarantula Toxin. Mol. Cell 81, 38–e4. 10.1016/j.molcel.2020.10.039 33232657 PMC8043720

[B8] Xu H. Li T. Rohou A. Arthur C. P. Tzakoniati F. Wong E. (2019). Structural Basis of Nav1.7 Inhibition by a Gating-Modifier Spider Toxin. Cell 176, 702–715. 10.1016/j.cell.2018.12.018 30661758

[B9] Zhao Y. Huang G. Wu J. Wu Q. Gao S. Yan Z. (2019). Molecular Basis for Ligand Modulation of a Mammalian Voltage-Gated Ca(2+) Channel. Cell 177, 1495–1506. e1412. 10.1016/j.cell.2019.04.043 31150622

[B10] Zimmer T. Haufe V. Blechschmidt S. (2014). Voltage-Gated Sodium Channels in the Mammalian Heart. Glob. Cardiol. Sci. Pract. 2014, 449–463. 10.5339/gcsp.2014.58 25780798 PMC4355518

